# Changing the Patient’s Position: Pitfalls and Benefits for Radiation Dose and Image Quality of Computed Tomography in Polytrauma

**DOI:** 10.3390/diagnostics12112661

**Published:** 2022-11-02

**Authors:** Jessica Graef, Keno K. Bressem, Patrick Asbach, Bernd Hamm, Stefan M. Niehues

**Affiliations:** 1 Department of Radiology, Campus Benjamin Franklin, Charité—Universitätsmedizin Berlin, 12203 Berlin, Germany; 2 Berlin Institute of Health at Charité—Universitätsmedizin Berlin, 10117 Berlin, Germany

**Keywords:** computed tomography, polytrauma, patient’s positioning, radiation exposure, image quality, iterative reconstruction

## Abstract

For computed tomography (CT), representing the diagnostic standard for trauma patients, image quality is essential. The positioning of the patient’s arms next to the abdomen causes artifacts and is also considered to increase radiation exposure. The aim of this study was to evaluate the effect of various positionings during different CT examination steps on the extent of artifacts as well as radiation dose using iterative reconstruction (IR). 354 trauma-CTs were analyzed retrospectively. All datasets were reconstructed using IR and three different examination protocols were applied. Arm elevation led to a significant improvement of the image quality across all examination protocols (*p* < 0.001). Variation in arm positioning during image acquisition did not lead to a reduction of radiation dose (*p* = 0.123). Only elevation during scout acquisition resulted in the reduction of radiation exposure (*p* < 0.001). To receive high-quality CT images, patients should be placed with elevated arms for the trunk scan, as artifacts remain even with the IR. Arm repositioning during the examination itself had no effect on the applied radiation dose because its modulation refers to the initial scout obtained. In order to achieve a dose effect by different positioning, a two-scout protocol (dual scout) should be used.

## 1. Introduction

Several technical innovations in the field of multi-detector computed tomography (CT) during the past 20 years have resulted in an enormous improvement in image acquisition, reconstruction time, and image quality due to minimization of motion and pulsation artifacts, as well as in reduction of radiation exposure [1,2,3,4,5,6,7]. Therefore, computed tomography could be implemented effectively in emergency care and it has proved to be a factor in reducing mortality in polytrauma patients [8,9,10,11,12,13]. Today, whole-body-CT (WBCT) represents the gold standard for diagnostics in the primary survey of severely injured patients [14,15,16,17]. According to the recent literature, 76% of patients suspected to be suffering from major trauma or polytrauma receive a WBCT in German emergency departments [18]. Due to the lack of a standardized examination protocol for trauma CTs, the radiation dose ranges from 10 mSv to 23 mSv [19,20,21,22]. This is especially relevant for young patients affected by polytrauma. The used protocols also differ concerning acquired examination sequences, included body regions, contrast media application technique, and examination time [15,22,23,24,25,26]. Thus, several publications raise the issue of an optimized procedure. The primary goals are the improvement of image quality and the reduction of radiation dose.

The patient’s positioning, especially arm positioning, has been proved to influence image quality in CT using filtered back projection (FBP); beam hardening artifacts caused by the bones of the upper extremity when placed adjacent to the abdomen affect the image quality of the parenchymatous organs to variable degrees [22,23,24,27]. In addition, several studies show a significant reduction of the radiation exposure when the patient is positioned with elevated arms [22,24,28]. For this reason, numerous hospitals have decided to reposition trauma patients between the different phases of a CT examination. The effects of the iterative reconstruction (IR) have been examined in emergency diagnostics especially with regard to reducing the radiation dose while maintaining or improving the image quality. 

The aim of this study was to evaluate the effect of the patient’s arm positioning in elevation or adduction on image quality and radiation dose during different steps of the CT protocol while using iterative reconstruction techniques. 

It was assumed that adducted arms required a higher radiation dose in order to scan the additionally radiographed tissue of the upper extremity, and that the image quality was likely to deteriorate due to resulting beam-hardening artifacts despite the application of IR. Patients receiving a CT examination with elevated arms should present with better image quality and decreased radiation dose accordingly.

## 2. Materials and Methods

This study has been approved by the local ethics committee (approval number EA4/140/17). The patients included were not exposed to additional radiation.

### 2.1. Patients’ Characteristics

Between 1 February 2018 and 6 June 2019, 283 CT examinations of severely injured patients (172 males, 111 females, mean age 55 years), performed for the primary survey in the emergency department of two German level-one trauma centers, were included in this study according to the following criteria: the patient received a contrast-enhanced thoracoabdominal CT examination during the initial evaluation of polytrauma patients, and both arms were either elevated above the head (n = 168, protocol B) or placed next to the abdomen (n = 115, protocol A) during the generation of the diagnostic images. Patients settled with arms in different positions or receiving a WBCT including the lower extremities were excluded. 

For comparing a third protocol, protocol C, 71 patients (48 males, 23 females, mean age 52 years), who underwent WBCT between 1 May 2021 and 31 May 2021, were included. All in- and exclusion criteria mentioned before were met by this study group accordingly. 

**Figure 1:**
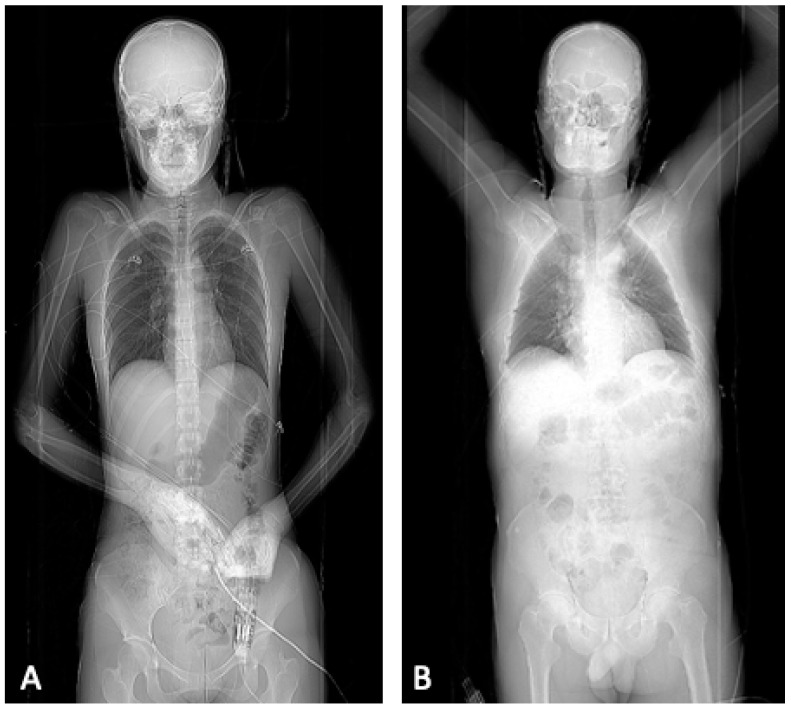
Example for the different positions of the patient during the scout varying between the evaluated computed tomography protocols. Image (**A**) shows the scout obtained for protocol A and B, whereas image (**B**) displays the scout of protocol C.

The images for protocol A and B were generated by using the following internal standardized protocol for severely injured patients (Figure 1):

1. Single whole-body scout, both arms adducted
2. Non-enhanced head CT, both arms adducted
3. CT-angiography of the head and neck, both arms adducted
4. Contrast-enhanced examination of the thorax and abdomen using
  ○ Protocol A: without repositioning, both arms still in adduction
  ○ Protocol B: relocation and positioning of both arms in elevation

Protocol C added a second scout to the examination protocol:

1. First scout: head scout, both arms adducted
2. Non-enhanced head CT, both arms adducted
3. Second scout: whole-body scout, both arms elevated
4. CT-angiography of head and neck in elevation
5. Contrast-enhanced examination of thorax and abdomen in elevation

The relocation of the arms was adapted to the patient’s condition; if any contraindications for the elevation of the arms were clinically suspected, e.g., a plexus lesion or a fracture of any part of the shoulder girdle, the patient received the entire CT examination with adducted arms as referred to as protocol A. Protocol A without repositioning was also applied when the patient was hemodynamically unstable.

If no injury contradicting the elevation was suspected, the CT examination was carried out using protocol B in 2019 or protocol C in 2021, since a reduction in radiation exposure due to the arm elevation was suspected. Therefore, the patients were assigned to the protocols depending on their clinical condition as a main priority and on the reduction of radiation exposure as a secondary priority. 

The examinations were performed by different CT-scanners: two 64-detector-CT-scanners (Revolution HD or Revolution EVO, GE Healthcare, Chicago, IL, U.S.A.) and one 80-detector-CT-scanner (Aquilion PRIME, Canon Medical Systems, Otawara, Japan). As a contrast agent, 140 mL of iodinated contrast media (Xenetix 350, Guerbet, Villepinte, France) per patient were used in the split-bolus-technique.

The following data were collected: age and sex of the patient, applied radiation dose in mGy*cm and position of the patient during the initial scout and thoracoabdominal CT examination. All data referring to the CT images were obtained from the thoracoabdominal scans and all included CT examinations were stored and saved in the local Picture Archiving and Communication System (PACS). 

### 2.2. Image Quality

**Figure 2:**
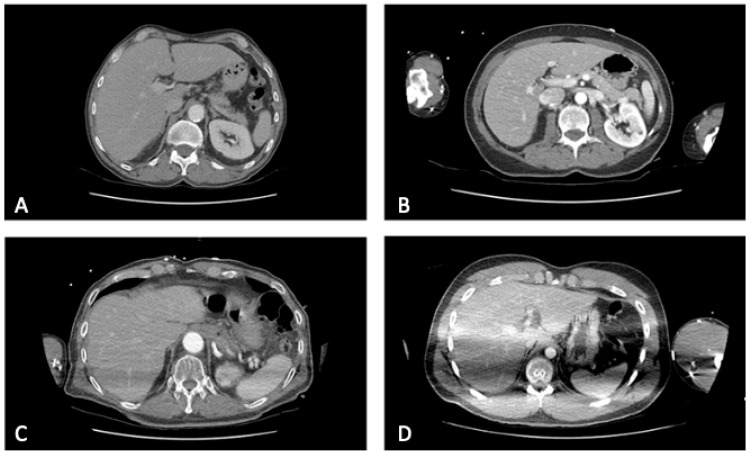
Axial contrast-enhanced CT images of the upper abdomen representing examples of the evaluation of the subjective image quality: (**A**) no artifacts resulting from the arms (score = 1), (**B**) artifacts without impairment of the image quality (score = 2), (**C**) artifacts with a moderate impairment (score = 3), (**D**) massive artifacts (score = 4).

A classification was developed in order to subjectively assess the image quality of the thoracoabdominal scans. A four-point-Likert-scale allowed a division in four different groups according to image quality determined by the severity and extent of the artifacts avoiding the option to choose a mean value (Figure 2).

1. = no artifacts
2. = artifacts without impairment of the image quality
3. = artifacts with a moderate impairment of the image quality
4. = artifacts with a massive impairment of the image quality

Artifacts caused by ECG-wires or implants, such as endoprostheses or pacemakers, were not considered as artifacts that could be altered by the positioning and therefore were not included in the assessment. 

Following an initial training period, the evaluation of the images, which were displayed to the analysts chronologically, was carried out by a senior radiologist reader with 16 years of experience in CT diagnostics and a second reader with more than one year of experience in the evaluation of trauma-CTs. For the analysis of image quality the focus was set on the display of the parenchymatous organs in the upper abdomen of the venous phase with 5 mm slice thickness.

### 2.3. Radiation Dose

The estimated radiation dose of the thoracoabdominal scans and the dose-length product (DLP) in mGy*cm were determined in all cases. The DLP, provided in the dose report of the CT scanner, was taken to compare the radiation doses between the different CT protocols evaluated.

### 2.4. Statistical Analysis

The normally distributed variables were displayed with mean ± standard deviation and compared with the independent *t*-test with a confidence level of 95%. The minimum number of 377 included patients was exceeded in order to reach meaningful statistical results (confidence level of 0.95 and α of 0.05). Non-normally distributed variables were displayed by median and interquartile ranges and compared with the Mann-Whitney U test, which was also used for comparing the Likert scales. A *p*-value of *p* ≤ 0.05 was considered statistically significant. All used charts were generated with SPSS (SPSS^®^, v. 25.0; IBM Corp., Armonk, NY, USA).

## 3. Results

An overview of the patients’ characteristics is shown in Table 1. The groups varied significantly concerning the artifacts determining image quality, radiation dose, and patients’ age. 

### 3.1. Image Quality

**Figure 3:**
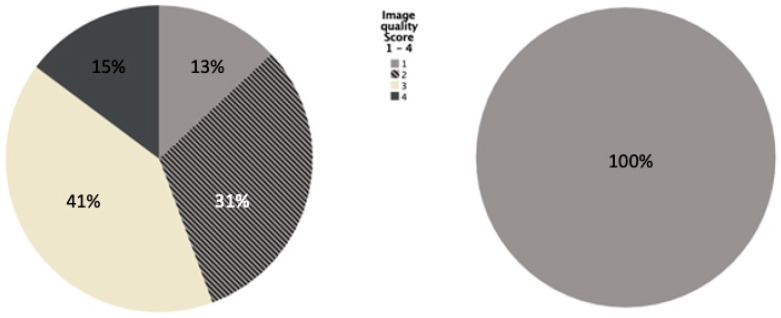
Rating of the image quality displayed by the different protocols (*p* < 0.001). When the examination is carried out in elevation in the protocols B and C, no artifacts from the upper extremities occur and all images are rated with score 1.0 (**right**). When the arms are placed next to the abdomen in protocol A, artifacts occur to a variable degree (**left**). The extent of artifacts is represented in percentages by the different colors according to the legend included in the figure.

As far as image quality is concerned, there was a statistically significant difference between the positioning options of protocol A and B (*p* < 0.001). Eighty-seven percent of the CT images of patients with adducted arms showed artifacts to a variable degree, of which 47% were moderately affected by artifacts. CT examinations of patients positioned with elevated arms presented with a significantly better image quality. For protocol B and C, which were both carried out in elevation, 100% of the CTs showed no artifacts resulting from arms or shoulder girdle and therefore the average value equals 1.0 (Figure 3). 

### 3.2. Radiation Dose

**Figure 4:**
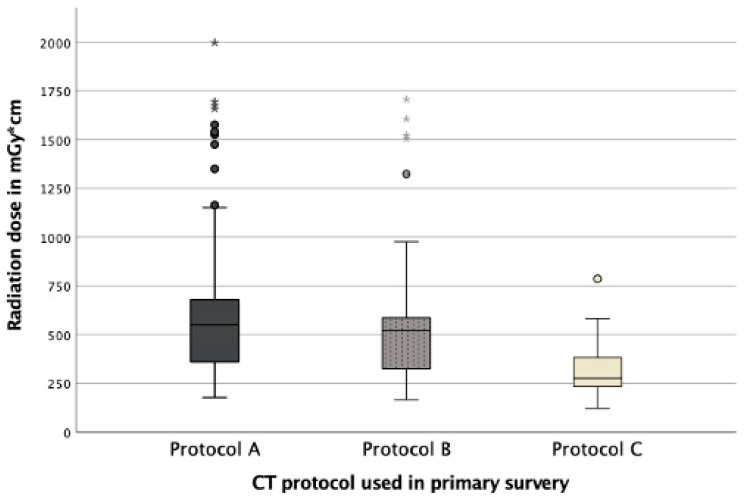
Comparison of medians and interquartile ranges of radiation dose depending on the CT protocol applied. No difference between the radiation doses of protocol A and B could be detected (*p* = 0.123), but between protocols A and B compared to protocol C (*p* < 0.001). The circles and stars (*) refer to radiation doses outside the interquartile ranges.

The applied radiation dose did not show a significant difference (*p* = 0.123) concerning the positioning of the patient during the CT examination of the abdomen. Patients with adducted arms received a median of 550 mGy*cm and patients with arms in an elevated position a median of 521 mGy*cm. Patients who underwent a CT using protocol C received a significantly lower radiation dose of 276 mGy*cm (*p* < 0.001) (Figure 4). 

## 4. Discussion

This study confirms a significant improvement of image quality when arms are elevated. With adducted arms relevant artifacts occur despite the use of modern IR. However, contrary to the literature, it could not be confirmed that the elevation of arms during image acquisition automatically leads to a reduction of radiation dose. This benefit can only be achieved by placing the arms in elevation for the scout as described in protocol C. After evaluating the results of this study, protocol C was implemented as the second, internal hospital standard protocol in addition to protocol A, depending on the patient’s condition for reducing the radiation exposure of trauma patients in this institution.

### 4.1. Image Quality

While positioning patients with elevated arms, the image quality of the CTs in this study could be improved significantly by reducing beam-hardening artifacts, generated by the higher density of the bones in the upper extremities resulting in an increased diffusion of photons.

With respect to the image quality determined by artifacts, the presented study has confirmed the results from current publications. Karlo et al. showed that the image quality of CTs of the organs in the upper abdomen as well as the spine is significantly better when the patient’s arms are elevated [22]. Furthermore, Karlo et al. stated that if the elevation of a patient’s arms is not possible, a cushion can be placed between the flexed arms and the patient’s chest in order to also improve the image quality in comparison to adducted arms [22]. Compared to the image quality in examinations with elevated arms, placing arms on a pillow reduces the image quality of the parenchymatous organs in the abdomen [25].

Positioning of only one arm in elevation does represent an alternative; according to Kahn et al. it also improves the image quality significantly compared to the positioning of both arms in adduction [23].

Aside from the improvement of image quality by arm elevation, Bayer et al. stated that the duration of the examination between the positions in elevation and adduction do not differ significantly [28]. Using a pillow and therefore refraining from changing the patient’s position shortens the examination time significantly, as shown by Hickethier et al. [25].

Geyer et al. described a reduction of the applied dose and an improved image quality by using IR in comparison to FBP [29]. According to Kahn et al. a 23% reduction of radiation dose is possible while maintaining equivalent image quality by using IR compared to FBP [21]. The data were obtained in the period from 2008 to 2013, so that since then improvements have been achieved in IR and it was possible to evaluate this improved influence by IR on artifacts in this study. It revealed that the positioning of the patient, despite all changes and new developments in reconstruction techniques, still plays a significant role in determining image quality.

### 4.2. Radiation Dose

Regarding the radiation dose no difference could be detected between the two positions during the thoracoabdominal CT examination itself (protocol A and B). Only protocol C, using a scout in elevation, allowed a dose reduction of 47.5% compared to protocol A and B.

The above-mentioned study by Karlo et al. presented a radiation dose reduction from 21.2 mSv to 16.1 mSv with arms in elevated position [23]. The detailed examination protocol is provided in that study without reference to the scouts. According to the included images, a scout in elevation was obtained for planning the examination.

Bayer et al. also achieved a dose reduction of 22% by placing the patient’s arms in elevation for the trunk scans. Their protocol uses a scout in elevation and therefore, the findings presented in this study come to the same conclusion.

Brink et al. stated an increase of the effective radiation dose by 45% for the positioning of patients with arms next to the abdomen [24]. Since the examination protocol is not provided, further comparison is not possible. It can be assumed that Brink et al. applied the same technique by using a scout in elevation in order to reduce the radiation exposure.

Depending on the technology applied in the emergency departments in this study, the radiation dose is calculated by the scout obtained at the beginning of the examination. Since head and neck CT scans are taken first, the scout is carried out with adducted arms. Due to the calculation from the scout, the repositioning of the patient during the thoracoabdominal CT does not result in any reduction of radiation dose.

A possibility to reduce the dose of radiation, as shown in this study by the application of protocol C, for patients without injuries of the shoulder girdle can be to perform two scouts (dual scout) in different parts of the examination. Nevertheless, the possible contraindications for the elevation of the arms should be evaluated carefully in each case in order to prevent iatrogenic injuries.

## 5. Limitations

The authors of this study could not be blinded to the position of the patients’ arms because of distinctive beam-hardening artifacts. The CT-scanners used in the participating emergency departments covered only two companies that manufacture CT-scanners worldwide (GE and Canon), and therefore, the findings presented in this study may not be transferable to all current CT scanners from other manufacturers.

## 6. Conclusions

Despite considerable progress in image reconstruction and artifact-reducing technique due to the implementation of the IR, arm positioning still has a major impact on the image quality. The initial protocol used in this study with elevating the arms after scout acquisition does not show an effect on the radiation dose, as the dose calculation depends on the initially obtained scout. A dual scout protocol was able to significantly reduce radiation dose by up to 47.5% while maintaining the image quality for the thoracoabdominal scans.

## Figures and Tables

**Table 1:** Overview of the patient characteristics divided by the three CT protocols examined.

	Protocol A	Protocol B	Protocol C	*p*-Value *	*p*-Value **
**Numbers** (n)	115	168	71		
**Sex** m/f	76 (66.09%)/ 39 (33.91%)	97 (57.40%)/ 72 (42.60%)	48 (67.6%)/ 23 (32.4%)	0.131	0.290
**Age** mean (age range)	58 (14–97)	52 (13–97)	52 (19–91)	0.019	0.308
**Artifacts** mean ± SD	2.6 ± 0.9	1.0 ± 0	1.0 ± 0	<0.001	<0.001
**Radiation dose** in mGy*cm median (minimum–maximum)	550 (178–1997)	521 (165–1706)	276 (122–787)	0.123	<0.001

* Differences in characteristics between protocol A and B, ** differences in characteristics between protocol A and B combined and protocol C. Overview of patient characteristics with both arms elevated above the head during the examination (protocol A), adducted next to the abdomen (protocol B) and with the scout and the examination in elevation (protocol C). The protocols A and B differ concerning the image quality (*p* < 0.001) and age (*p* = 0.019), whereas protocol C differs from A and B combined in image quality (*p* < 0.001) and radiation exposure (*p* < 0.001).

## Data Availability

The data presented in this study are available upon request from the corresponding authors.
